# Deep learning–accelerated contrast-enhanced 3D MRI for acute optic neuritis: faster acquisition and improved lesion conspicuity

**DOI:** 10.3389/fneur.2026.1954788

**Published:** 2026-10-15

**Authors:** Ying Liu, Weiyin Vivian Liu, Zhi Wen, Min Lu, Changsheng Liu, Liang Li, Ting Chen, Renjie Yang, Yunfei Zha

**Affiliations:** 1 Department of Radiology, Renmin Hospital of Wuhan University, Wuhan, Hubei, China; 2 MR Research, GE Healthcare, Beijing, China; 3 Department of Ophthalmology, Renmin Hospital of Wuhan University, Wuhan, Hubei, China

**Keywords:** contrast-enhanced T1-weighted imaging, deep learning reconstruction, MRI acceleration, optic neuritis, orbital magnetic resonance imaging

## Abstract

**Objective:**

To compare a deep learning-accelerated contrast-enhanced 3D T1-weighted sequence (T1-CUBE-DLS) with standard parallel imaging-accelerated T1-CUBE for detecting acute optic neuritis (ON).

**Methods:**

This prospective study enrolled 59 patients with suspected acute ON (38 females; mean age 41.2 ± 12.8 years). All underwent contrast-enhanced whole-brain 3.0 T MRI including both T1-CUBE-DLS and standard T1-CUBE sequences. Two blinded neuroradiologists independently scored motion artifacts, subjective noise, and optic nerve clarity. Consensus readings recorded enhancement laterality, involved segments, and visual conspicuity. Enhancement length was measured per optic nerve, with inter-observer reliability assessed via intraclass correlation coefficient (ICC) and Bland–Altman analysis. Clinical diagnosis by a senior neuro-ophthalmologist served as the reference standard.

**Results:**

Acute ON was confirmed in 43 of 59 patients (72.9%). T1-CUBE-DLS reduced acquisition time by 36.2%. Subjective noise was equivalent between sequences (*p* > 0.05), but T1-CUBE-DLS showed significantly fewer motion artifacts and superior optic nerve clarity (both *p* < 0.05). No significant difference was detected in diagnostic performance between the two sequences: sensitivity (88.4% vs. 86.0%), specificity (93.8% vs. 93.8%), and accuracy (89.8% vs. 88.1%; all *p* > 0.05). T1-CUBE-DLS demonstrated stronger visual conspicuity (*p* = 0.011) and slightly longer measured enhancement length (mean difference, 0.836 mm; *p* < 0.0001). ICC for length measurement was 0.91 for T1-CUBE-DLS vs. 0.83 for standard T1-CUBE, with narrower 95% limits of agreement (−1.37 to 1.51 mm vs. − 3.1 to 2.4 mm).

**Conclusion:**

T1-CUBE-DLS reduces acquisition time by 36.2%, with no significant difference in diagnostic accuracy detected, while improving enhancement conspicuity and measurement reproducibility, supporting its potential as a practical and efficient alternative for ON evaluation.

## 1 Introduction

Acute optic neuritis (ON) is an inflammatory demyelinating disease affecting the optic nerve, frequently leading to transient or permanent visual dysfunction (1, 2). It may present as an isolated clinical event or occur secondary to systemic demyelinating syndromes, including multiple sclerosis (MS), neuromyelitis optica spectrum disorder (NMOSD), and MOG antibody-associated disease (MOGAD) (3). Core clinical manifestations include acute unilateral vision loss, visual field deficits, dyschromatopsia, and retro-orbital pain exacerbated by eye movement (4–6).

Standard diagnostic workflows typically combine clinical history, neuro-ophthalmic evaluation (including visual evoked potentials, optical coherence tomography, and fundoscopy), and orbital MRI to confirm ON and facilitate differential diagnosis of other visual pathway pathologies (7, 8). Recent MAGNIMS guidelines emphasize the diagnostic value of contrast-enhanced T1-weighted imaging (CE-T1WI) of the orbit in the evaluation of suspected optic neuritis (9). Optic nerve enhancement visualized on contrast-enhanced T1WI is highly sensitive for active demyelination; moreover, the anatomical location and longitudinal length of enhancing segments strongly correlate with long-term visual recovery and therapeutic response (10–12). Conventional 2D fast spin echo (FSE) CE-T1WI suffers from prominent partial-volume averaging and vascular pulsation artifacts (13). 3D FSE sequences deliver superior spatial resolution and mitigate partial-volume effects, enabling visualization of small orbital anatomical structures (14, 15). However, the extended scan time of standard 3D FSE limits widespread clinical adoption (16, 17).

Traditional acceleration strategies including parallel imaging (PI) and compressed sensing rely on sparse k-space sampling, which introduces amplified noise and geometric artifacts at high acceleration factors, degrading diagnostic image quality (18–20). DL-Speed (DLS) is a commercially available deep learning reconstruction framework optimized for rapid MRI acquisition. It reconstructs highly undersampled multi-coil k-space data via unrolled optimization networks with dense skip-layer connections and embedded data consistency constraints, effectively suppressing noise while preserving anatomical edge definition (21). Prior brain and liver MRI work confirmed DLS yields superior image quality and shorter scan times for 3D acquisitions compared to conventional PI (22). Our 3D FSE sequence of interest was whole-brain T1-CUBE, a commercially available isotropic volumetric sequence. No prospective study has compared DLS-accelerated T1-CUBE with standard PI-accelerated T1-CUBE in acute ON. The present study addresses this gap by evaluating image quality, optic nerve enhancement features, and diagnostic performance, with secondary assessment of inter-reader measurement reproducibility.

## 2 Methods

### 2.1 Study population

This prospective observational comparative study received formal approval from the institutional Clinical Ethics Committee (IRB No. WDRY2023-Y136), with all procedures performed in strict accordance with the Declaration of Helsinki. Consecutive patients presenting with high clinical suspicion of acute ON between May and November 2025 were screened for enrollment. Written informed consent was obtained from all participants; for participants under 18 years, consent was provided by a parent or legal guardian. The definitive reference standard diagnosis of acute ON was rendered by a senior neuro-ophthalmologist after comprehensive integrated assessment, including: 1. Clinical symptomatology (acute/subacute monocular vision loss, movement-evoked ocular pain, relative afferent pupillary defect, color desaturation); 2. Complete neuro-ophthalmic workup (fundoscopy, visual evoked potentials, optical coherence tomography); 3. Clinical follow-up to exclude alternative ocular and intracranial pathologies. The neuro-ophthalmologist had access to the written MRI reports, but did not review the MRI images directly.

Inclusion criteria: 1. No absolute contraindications to 3.0 T MRI; 2. No documented gadolinium-based contrast medium allergy; 3. Non-pregnant female patients of childbearing age. Exclusion criteria: 1. Confirmed primary visual pathway lesions (pituitary macroadenoma, optic nerve meningioma, orbital mass); 2. Inability to complete full MRI acquisition due to severe claustrophobia or involuntary movement; 3. Severe ferromagnetic foreign body artifacts precluding reliable orbital evaluation.

Initially, 66 patients were screened. Seven patients were excluded (5 with optic pathway tumors, 1 failed T1-CUBE-DLS acquisition, 1 severe metal artifact), yielding a final cohort of 59 subjects (38 female [64.4%], 21 male [35.6%]; mean age 41.2 ± 12.8 years, range 16–62 years).

### 2.2 MRI acquisition protocol

All examinations were performed on a 3.0 T SIGNA Architect MRI system (GE Healthcare, United States) equipped with a 48-channel head coil. Pre-contrast localizer sequences included axial 2D T1WI, fat-suppressed T2WI, and coronal short-tau inversion recovery. After the intravenous administration of a standard dose (0.2 mL/kg; 0.1 mmol/kg) of gadodiamide (Omniscan®, GE Healthcare), two whole-brain sagittal 3D T1-CUBE sequences were acquired: 1. T1-CUBE-DLS: DLS acceleration factor = 9; 2. Standard T1-CUBE: PI acceleration factor = 4. The acquisition order was alternated across the cohort to mitigate potential bias from contrast timing: T1-CUBE-DLS was acquired first in 31 patients, and standard T1-CUBE first in the remaining 28 patients. The first post-contrast sequence was initiated approximately 2 min after injection, and the second post-contrast sequence was acquired immediately after the first. Identical acquired voxel size (1 × 1 × 1 mm^3^) was used for both sequences; zero-filling interpolation in the vendor reconstruction pipeline generated reconstructed isotropic 0.5 × 0.5 × 0.5 mm^3^ voxels for multiplanar reformation (MPR). Full standardized imaging parameters are summarized in Table 1.

**Table 1:** Imaging acquisition parameters.

Items	Standard T1-CUBE	T1-CUBE-DLS
Sequence type	FSE	FSE
Acquisition mode	3D	3D
Acquisition plane	Sagittal	Sagittal
TR/TE (ms)	500/15	500/15
No. of excitations	1	1
Echo-train length	24	24
Field of view (mm)	240	240
Matrix	240 × 240	240 × 240
Acquired voxel size (mm^3^)	1 × 1 × 1	1 × 1 × 1
Reconstructed voxel size (mm^3^)	0.5 × 0.5 × 0.5	0.5 × 0.5 × 0.5
Acceleration technique	Parallel imaging	DL-Speed
Acquisition time (min)	03:44	02:23

FSE = fast spin-echo; TR = repetition time; TE = echo time.

DLS (GE Healthcare) framework is a vendor-provided, pre-trained DL-based acceleration technique based on an unrolled optimization architecture with dense skip-layer connections. It reconstructs complex-valued MR images from highly undersampled multi-coil k-space data by iteratively applying a data consistency unit and a convolutional neural network (CNN)-based regularization unit (22).

### 2.3 Qualitative image quality assessment

Two independent radiologists (Observer 1 and Observer 2, with 8 and 6 years of neuroradiology experience, respectively), both blinded to all clinical and sequence information, reviewed the two MRI datasets on a diagnostic workstation. Prior to the formal assessment, both observers participated in a calibration session using ten sample cases (not included in the study cohort) to reach consensus on the interpretation of the scoring scales.

To reduce recall bias, a crossover reading design with two distinct sessions, independent of the sequence acquisition order, was employed (Figure 1). The 59 patients were randomly divided into two groups (Group 1, *n* = 30; Group 2, *n* = 29). In Session 1, both observers evaluated T1-CUBE-DLS images from Group 1 and standard T1-CUBE images from Group 2. After a two-week washout interval, Session 2 was performed with reversed sequence assignments: both observers evaluated standard T1-CUBE images from Group 1 and T1-CUBE-DLS images from Group 2. Within each session, the case presentation order was fully randomized. All patient identifiers and sequence labels were removed.

**Figure 1:**
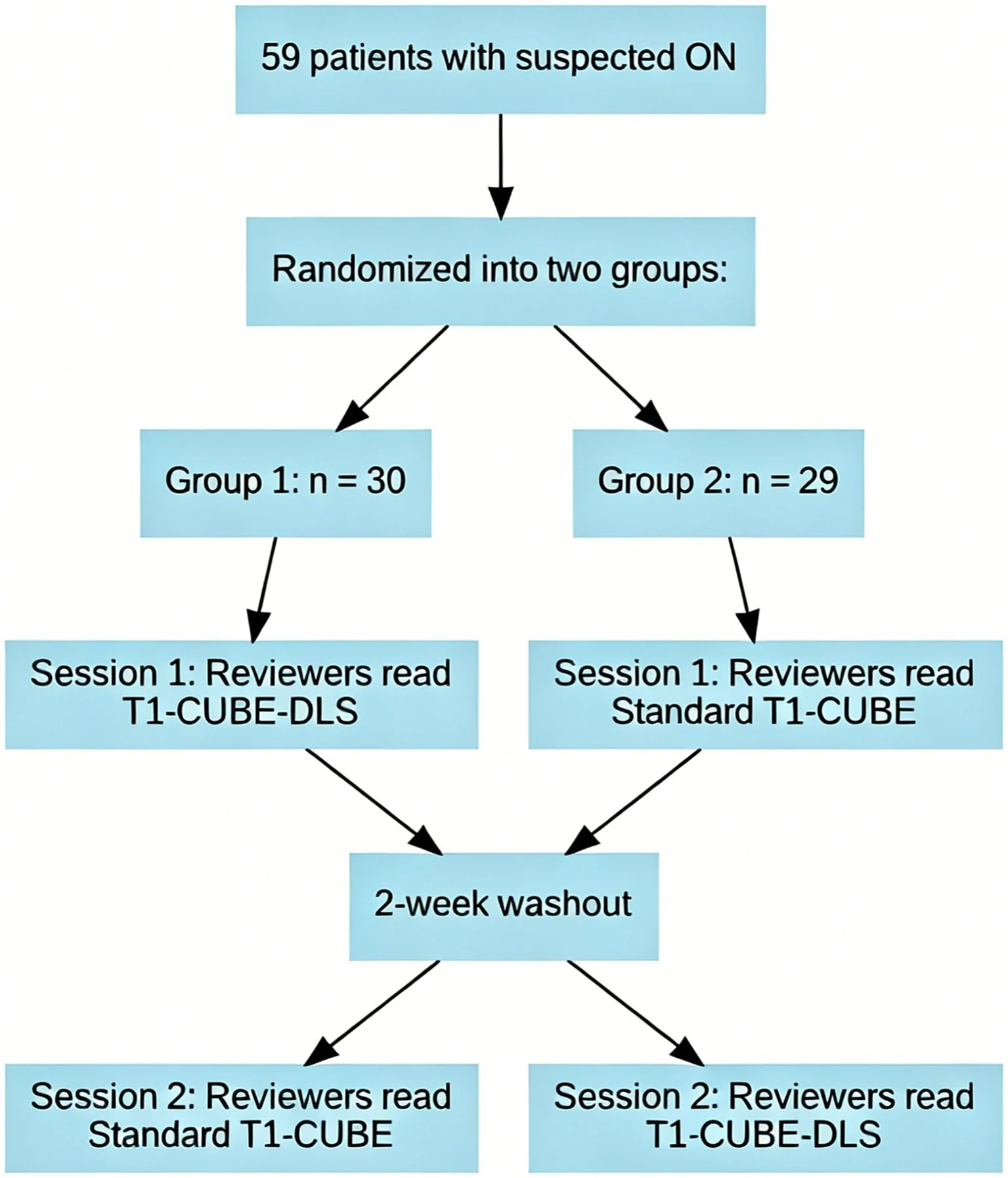
Crossover blinded reading workflow flowchart. Fifty-nine patients were randomly divided into Group 1 (*n* = 30) and Group 2 (*n* = 29). In Session 1, readers evaluated T1-CUBE-DLS for Group 1 and standard T1-CUBE for Group 2. After a 2-week washout interval to reduce recall bias, Session 2 reversed the sequence assignments. All patient identifiers and sequence labels were removed during blinded evaluation.

The following qualitative parameters were scored using predefined 3 or 4-point scales: 1. Motion artifacts (3-point scale): 3 = minimal/no motion artifacts; 2 = motion artifacts present but not interfering with optic nerve delineation; 1 = motion artifacts impairing delineation. 2. Subjective noise (3-point scale): 3 = minimal or no noise; 2 = noise present but not interfering with delineation of the optic nerve; 1 = noise impairing delineation. 3. Optic nerve clarity (4-point scale): 4 = excellent clarity with sharp borders; 3 = good clarity with mildly indistinct borders; 2 = moderate clarity with blurred borders; 1 = poor clarity with poorly defined borders.

### 2.4 Optic nerve enhancement analysis

Following independent qualitative scoring, the two observers reached a consensus evaluation for all enhancement-related endpoints on a clinical PACS workstation with standardized window/level settings. Orthogonal MPR reconstructions (0.5 mm slice thickness, no slice gap) aligned parallel and perpendicular to the long axis of each optic nerve were generated to optimize lesion visualization. Pre-contrast sequences were referenced to differentiate pathological contrast enhancement from physiological vascular signal.

Recorded enhancement features included: Presence or absence of optic nerve enhancement; Laterality (unilateral/bilateral); Involved segment (intraorbital, canalicular, intracranial); Visual conspicuity (slight, moderate, strong). Laterality was recorded at the patient level. Involved segment and visual conspicuity were assessed at the patient level; in bilateral cases, the more conspicuous affected nerve was used. These assessments were restricted to ON-positive patients with confirmed enhancement.

For enhancement length, the unit of analysis was the individual optic nerve. Among ON-positive patients with confirmed enhancement on both sequences, all affected nerves were measured independently. Enhancement length was measured independently by each observer along the longitudinal axis of the optic nerve on reformatted images, from the proximal to the distal margin of enhancement, following the method described by Shaw et al. (12).

### 2.5 Statistical analysis

All statistical analyses were performed using MedCalc v11.6 (MedCalc Software, Ostend, Belgium). A two-tailed *p* < 0.05 was defined as the threshold for statistical significance. Ordinal image quality scores between sequences were compared via paired Wilcoxon signed-rank tests; inter-reader agreement for qualitative scores was quantified with weighted Cohen’s kappa (κ): poor <0.20, fair 0.21–0.40, moderate 0.41–0.60, good 0.61–0.80, excellent 0.81–1.00. Binary enhancement detection and laterality were compared using McNemar exact test. Because a single patient could have multiple segments involved, involved segment was analyzed as three separate binary variables (intraorbital, canalicular, and intracranial); McNemar exact test was applied to each segment with Bonferroni correction for multiple comparisons (adjusted significance threshold, *p* < 0.0167). Visual conspicuity was compared using McNemar-Bowker test; cell-wise comparisons were *post hoc* and adjusted for multiplicity using Bonferroni correction (adjusted significance threshold, *p* < 0.0167). Continuous enhancement length measurements were compared with paired Student’s *t*-test. Inter-observer quantitative reliability was assessed via ICC and Bland–Altman analysis to calculate 95% limits of agreement (LOA). Diagnostic sensitivity, specificity, and overall accuracy with 95% confidence intervals (Clopper-Pearson exact method) were calculated for each sequence against the neuro-ophthalmologist clinical gold standard.

## 3 Results

### 3.1 Patient baseline characteristics

Of the 59 enrolled patients, 43 (72.9%) received a confirmed clinical diagnosis of acute ON, while the remaining 16 (27.1%) served as non-ON controls, with final diagnoses of non-arteritic anterior ischemic optic neuropathy (*n* = 7), functional visual loss (*n* = 4), retinal disease (*n* = 4), and other non-inflammatory conditions (*n* = 1). Among ON-positive patients, underlying etiologies were classified as: idiopathic demyelinating ON (double-seronegative, *n* = 21, 48.8%), MS-associated ON (*n* = 12, 27.9%), NMOSD (*n* = 6, 14.0%), MOGAD (*n* = 4, 9.3%). Involvement was unilateral in 41 patients (95.3%) and bilateral in 2 (4.7%), corresponding to 45 total affected optic nerves (20 right, 25 left). Mean symptom duration prior to MRI scanning was 7.5 ± 2.6 days (range 1–14 days).

### 3.2 Qualitative image quality comparison

No significant difference in subjective noise was observed between the standard T1-CUBE and T1-CUBE-DLS sequences by either observer (*p* > 0.05). In contrast, both observers rated motion artifacts significantly lower and optic nerve clarity significantly higher on T1-CUBE-DLS compared to standard T1-CUBE (*p* < 0.05). A representative case is illustrated in Figure 2. Inter-observer agreement ranged from moderate to good for standard T1-CUBE (*κ* = 0.573–0.679) and from moderate to almost perfect for T1-CUBE-DLS (*κ* = 0.542–0.801). Detailed scores for the three image quality parameters are provided in Table 2.

**Figure 2:**
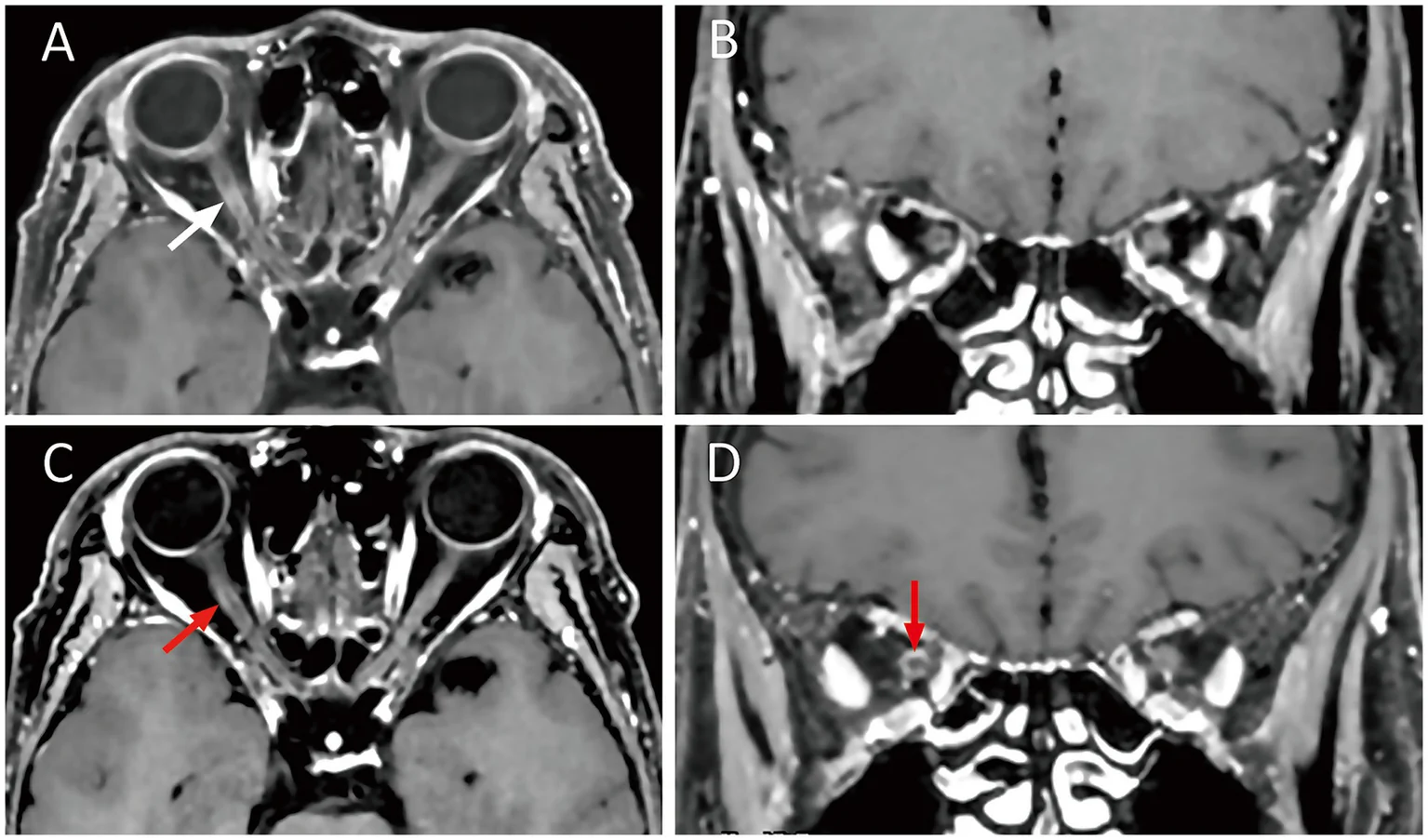
A 58-year-old woman with clinically confirmed right acute optic neuritis (symptom duration: 13 days). On the standard T1-CUBE sequence, slight enhancement is suggested but obscured by motion artifacts on the long-axis image [**(A)**, white arrow], with no definitive enhancement on the short-axis view **(B)**, leading to a consensus read of “no definite abnormality.” In contrast, the motion-robust T1-CUBE-DLS images clearly depict corresponding enhancement within the right optic nerve sheath in both the long-axis [**(C)**, red arrow] and short-axis [**(D)**, red arrow] planes, yielding a positive finding.

**Table 2:** Comparisons of image quality scores between standard T1-CUBE and T1-CUBE-DLS images.

Items	Observer	Standard T1-CUBE	T1-CUBE-DLS	*p-*value
Motion artifacts	1	2.492 ± 0.504	2.705 ± 0.460	0.0008*
	2	2.475 ± 0.503	2.672 ± 0.473	0.0005*
κ		0.573 (0.368–0.779)	0.542 (0.315–0.769)	
Subjective noise	1	2.623 ± 0.489	2.525 ± 0.504	0.2971
	2	2.508 ± 0.504	2.443 ± 0.501	0.2087
κ		0.607 (0.410–0.805)	0.801 (0.653–0.949)	
Optic nerve clarity	1	2.864 ± 0.629	3.356 ± 0.580	< 0.0001*
	2	2.898 ± 0.635	3.220 ± 0.559	0.0023*
κ		0.679 (0.512–0.846)	0.645 (0.460–0.830)	

The scores for qualitative items are expressed as mean ± standard deviation, with higher values indicating better outcomes. Motion artifacts and subjective noise were rated on a 3-point scale, and optic nerve clarity on a 4-point scale. Inter-observer agreement was assessed using weighted Cohen’s kappa (*κ*): poor (< 0.20), fair (0.21–0.40), moderate (0.41–0.60), good (0.61–0.80), and excellent (0.81–1.00). * indicates a statistically significant difference (*p* < 0.05).

### 3.3 Optic nerve enhancement and diagnostic performance

Consensus reading identified optic nerve enhancement in 38 cases on standard T1-CUBE and 39 cases on T1-CUBE-DLS. This included one false-positive case in a control patient with normal perineural venous plexus enhancement. In addition, one ON-positive case was exclusively visible on T1-CUBE-DLS due to reduced motion artifacts (Figure 2). The full 2 × 2 contingency tables are provided in Table 3, and diagnostic metrics with 95% confidence intervals are presented in Table 4. Paired comparison showed that both sequences were positive in 38 cases, standard T1-CUBE positive with T1-CUBE-DLS negative in 0 cases, standard T1-CUBE negative with T1-CUBE-DLS positive in 1 case, and both negative in 20 cases. McNemar exact test showed no significant difference in enhancement detection between the two sequences (*p* = 1.000).

**Table 3:** Contingency tables for optic nerve enhancement detection against the clinical reference standard.

Sequence	TP	FP	FN	TN
Standard T1-CUBE	37	1	6	15
T1-CUBE-DLS	38	1	5	15

The reference standard was the comprehensive clinical diagnosis established by a senior neuro-ophthalmologist. TP = true positive; FP = false positive; FN = false negative; TN = true negative.

**Table 4:** Diagnostic performance of standard T1-CUBE and T1-CUBE-DLS.

Metric	Standard T1-CUBE	95% CI	T1-CUBE-DLS	95% CI
Sensitivity	86.0% (37/43)	72.1–94.7%	88.4% (38/43)	74.9–96.1%
Specificity	93.8% (15/16)	69.8–99.8%	93.8% (15/16)	69.8–99.8%
Accuracy	88.1% (52/59)	77.1–95.1%	89.8% (53/59)	79.2–96.2%

95% CIs were calculated using the Clopper-Pearson exact method. CI = confidence interval.

Optic nerve enhancement features are summarized in Table 5. Laterality, involved segment, and visual conspicuity were assessed in ON-positive patients with confirmed enhancement (*n* = 37 for standard T1-CUBE and *n* = 38 for T1-CUBE-DLS); the remaining ON-positive patients (*n* = 6 and *n* = 5, respectively) had no detectable enhancement and were excluded from these analyses. No significant differences were found between sequences regarding laterality or the distribution of the involved segment of enhancement (e.g., intraorbital, canalicular, intracranial). However, T1-CUBE-DLS demonstrated a significantly higher proportion of cases rated as showing strong visual conspicuity compared with standard T1-CUBE (*p* = 0.011; Bonferroni-adjusted significance threshold, *p* < 0.0167). A representative case is illustrated in Figure 3.

**Table 5:** Comparison of optic nerve enhancement assessment between standard T1-CUBE and T1-CUBE-DLS.

Items	Standard T1-CUBE (*n* = 37)	T1-CUBE-DLS (*n* = 38)	*p-*value
Laterality (unilateral/bilateral)	35/2	36/2	0.978
Involved segment			
Intraorbital	36 (97.3%)	35 (92.1%)	1.000
Canalicular	9 (24.3%)	11 (28.9%)	0.625
Intracranial	9 (24.3%)	8 (21.1%)	1.000
Visual conspicuity			
Slight	7 (18.9%)	8 (21.1%)	0.783
Moderate	20 (54.1%)	11 (28.9%)	0.061
Strong	10 (27.0%)	19 (50.0%)	0.011*

Analyses included ON-positive patients with confirmed enhancement. Bilateral cases used the more conspicuous nerve for involved segment and visual conspicuity. Laterality and involved segment were tested with McNemar exact test; involved segment was analyzed as three separate binary variables with Bonferroni correction (*p* < 0.0167). Visual conspicuity was compared using McNemar-Bowker test, with post hoc cell-wise comparisons Bonferroni-adjusted (*p* < 0.0167). *p < 0.0167.

**Figure 3:**
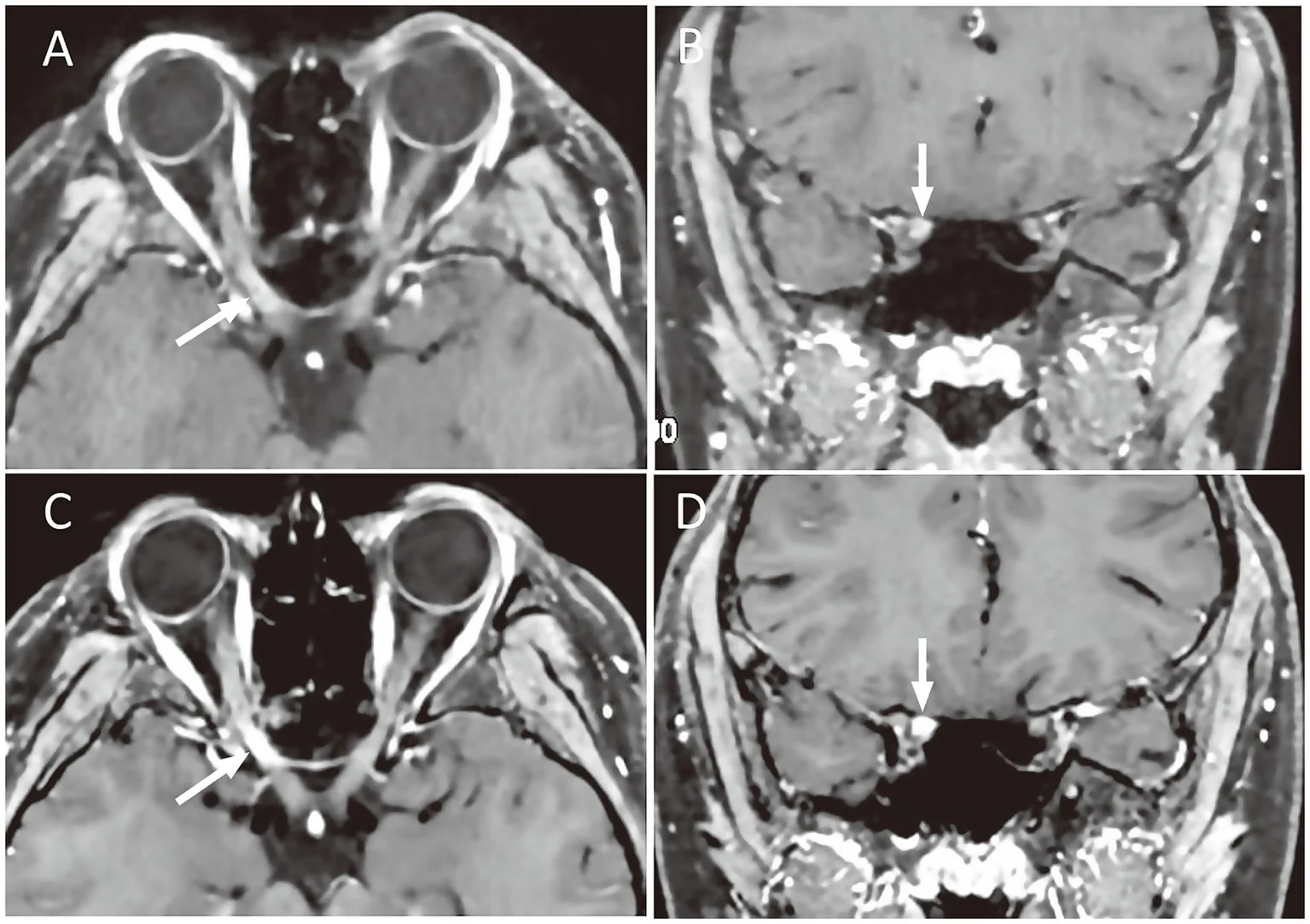
Representative MR images in a patient with right acute optic neuritis (4-day symptom duration). Compared to the standard T1-CUBE images **(A,B)**, the T1-CUBE-DLS images **(C,D)** show stronger visual conspicuity and clearer delineation of enhancement in the canalicular and intracranial segments of the right optic nerve (white arrow).

Enhancement length was measured in 39 optic nerves from 37 ON-positive patients (35 unilateral and 2 bilateral) with confirmed enhancement on both sequences. The measured enhancement length was greater on T1-CUBE-DLS than on standard T1-CUBE (17.093 ± 5.997 mm vs. 16.257 ± 5.663 mm; mean paired difference, 0.836 mm; 95% CI, 0.516–1.156 mm; *p* < 0.0001). Inter-observer ICC for enhancement length measurement was 0.91 for T1-CUBE-DLS and 0.83 for standard T1-CUBE. Bland–Altman analysis of inter-observer variability in length measurement revealed 95% LOA of −3.1 to 2.4 mm for standard T1-CUBE and −1.37 to 1.51 mm for T1-CUBE-DLS (Figure 4). The higher ICC and narrower 95% LOA for T1-CUBE-DLS indicate superior inter-observer measurement consistency compared with standard T1-CUBE.

**Figure 4:**
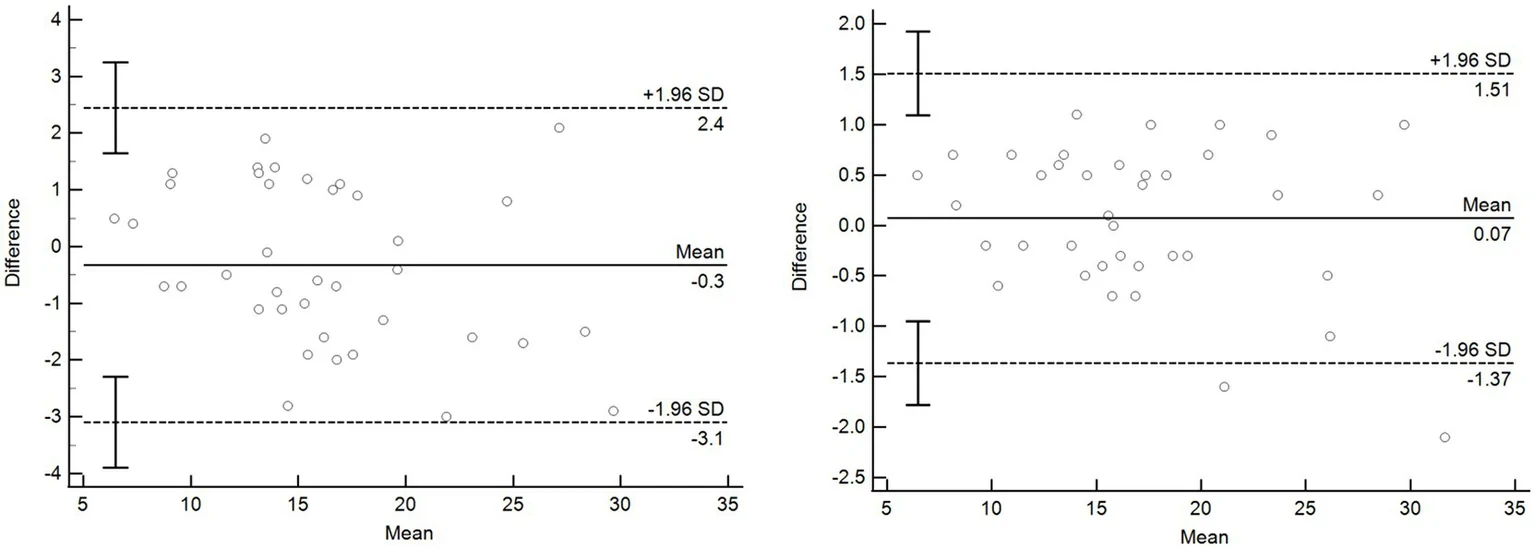
Bland–Altman plots for inter-observer differences of enhancement length measurement. The inter-observer 95% limits of agreement (LOA) were −3.1 to 2.4 mm for standard T1-CUBE (left) and −1.37 to 1.51 mm for T1-CUBE-DLS (right), indicating better agreement with the latter sequence.

## 4 Discussion

This prospective study compared T1-CUBE-DLS with standard T1-CUBE for the detection of acute ON. The results demonstrated that T1-CUBE-DLS, despite a 36.2% shorter acquisition time, showed no significant difference in diagnostic accuracy, with significantly lower motion artifacts, improved optic nerve clarity, stronger visual conspicuity, and superior inter-observer consistency in lesion measurement.

Acute ON poses unique imaging challenges stemming from the optic nerve’s narrow caliber, oblique anatomical course, and high susceptibility to eye motion and vascular pulsation, which frequently obscure subtle inflammatory enhancement (13, 16). Several strategies have been explored to address this challenge, each with distinct trade-offs.

The most direct approach has been the transition from 2D to 3D acquisition (14, 15). Yang et al. (15) reported higher diagnostic sensitivity for acute ON with 3D T1-CUBE (89%) relative to 2D T1WI (75%). Subsequent investigations by Prillard et al. (23) and Riederer et al. (24) further validated that whole-brain 3D T1WI achieves diagnostic accuracy comparable to dedicated orbital protocols, an advantage particularly relevant for patients screened for MS, NMOSD, and MOGAD. However, they consistently reported lower reader confidence on whole-brain images due to radiologists’ greater familiarity with orbital protocols. Our study, using T1-CUBE-DLS which similarly covers the whole brain, achieved diagnostic performance (sensitivity 88.4%, specificity 93.8%, accuracy 89.8%) comparable to prior 3D studies. Notably, the improved optic nerve clarity and stronger conspicuity of enhancing lesions may help address the reader confidence gap, as clearer delineation of orbital anatomy could facilitate more confident interpretation without requiring additional dedicated orbital acquisitions.

A second strategy focuses on motion robustness. Radial k-space sampling gradient-echo protocols such as Radial-VIBE have been proposed to reduce motion sensitivity in orbital imaging (17), yet gradient-echo readouts produce prominent magnetic susceptibility artifacts at the skull base, optic canal, and paranasal sinuses—regions essential for evaluating canalicular and intracranial ON segments. In our study, the higher acceleration factor of T1-CUBE-DLS shortened the acquisition time, thereby reducing the window for motion during scanning. This likely accounts for the significantly lower motion artifacts observed on T1-CUBE-DLS. Because the two sequences differed in both acceleration factor and reconstruction algorithm, the relative contributions of DL reconstruction and shorter scan time to the improved optic nerve clarity cannot be fully separated. The significantly lower motion artifacts and higher optic nerve clarity on T1-CUBE-DLS (*p* < 0.05) demonstrate that DL-accelerated FSE acquisition offers another effective approach for motion suppression, without GRE’s susceptibility limitations.

A third strategy to improve lesion detection is to increase contrast on post-contrast images. Previous studies (14, 24) have used specialized flow-suppressed preparations, such as double inversion-recovery (DIR) black-blood and DANTE-prepared T1-SPACE, to improve background signal nulling and thereby increase lesion conspicuity. These techniques can improve background suppression, but they require custom scanner programming that is not universally available across clinical platforms. T1-CUBE-DLS, in contrast, does not require specialized sequence preparation. In addition, post-contrast delay is a known factor influencing lesion conspicuity (24). In our study, the acquisition order was alternated to mitigate timing-related bias. Under these conditions, T1-CUBE-DLS showed significantly stronger visual conspicuity (*p* = 0.011), suggesting that DLS may represent an alternative approach to improving lesion conspicuity, possibly through the contrast-weighted loss optimization of its training framework (21, 25), without requiring specialized sequence preparation. However, direct head-to-head comparisons between DLS and these specialized techniques are needed.

A conceptually different approach to whole-brain assessment of the optic nerve was explored by Panyaping et al., who used CE 3D FLAIR with fat suppression to detect acute ON (26). FLAIR offers the theoretical advantage of combining T2 hyperintensity from edema with T1 shortening from enhancement, potentially increasing lesion conspicuity, with quantitative signal intensity ratio (SIR) cutoffs providing 93% sensitivity. While CE 3D FLAIR has merit, its interpretation is complicated by vascular enhancement artifacts and variable CSF suppression, and its reliance on SIR calculations adds complexity for routine use. In our study, T1-CUBE-DLS relies on standard CE-T1WI, consistent with routine clinical practice, and showed no significant difference in diagnostic performance relative to standard T1-CUBE, without requiring additional quantitative calculations (15). However, the specificity estimate should be interpreted with caution. Although the control group represented common differential diagnoses of suspected ON (e.g., non-arteritic anterior ischemic optic neuropathy, functional visual loss, and retinal disease), the small sample (*n* = 16) and limited diagnostic spectrum may restrict generalizability to the full range of differential diagnoses in clinical practice.

In addition, the clinical relevance of improved lesion measurement extends beyond diagnosis. Shaw et al. (12) reported that in aquaporin-4 (AQP4)-antibody-positive ON, an initial lesion length exceeding 18.21 mm predicted poor visual outcomes with 89.3% sensitivity, with involvement of the canalicular and intracranial segments also associated with worse prognosis. These observations underscore the clinical need for reliable lesion measurement. Our study addresses this by showing that T1-CUBE-DLS yielded slightly longer measured enhancement lengths than standard T1-CUBE (17.093 mm vs. 16.257 mm; mean paired difference, 0.836 mm; 95% CI, 0.516–1.156 mm; *p* < 0.0001). However, this 95% CI is relatively wide, indicating uncertainty in the true magnitude of the difference, and in the absence of an independent reference measurement, the direction of the bias cannot be determined. It may reflect more complete visualization of the true lesion extent, or alternatively, systematic overestimation due to edge smoothing. Given that a shift of approximately 0.8 mm could reclassify patients near the 18.21-mm prognostic threshold, the clinical significance of this small difference remains uncertain and warrants validation against a reference standard. Nevertheless, the improved inter-observer consistency [ICC 0.91 vs. 0.83, with narrower 95% LOA (−1.37 to 1.51 mm vs. − 3.1 to 2.4 mm)] supports more reproducible longitudinal monitoring of lesion length.

This study has several limitations. First, the sample size was relatively small, limiting the power to exclude a clinically relevant difference; larger studies with pre-specified non-inferiority margins are warranted. Second, because the two sequences differed in acceleration factor and acquisition time as well as reconstruction algorithm, the observed benefits reflect combined effects and cannot be attributed to DL reconstruction alone. Third, the reference diagnosis may have been influenced by the written MRI reports, potentially introducing incorporation bias; however, because both sequences were included in the reports, this bias likely affected them similarly, and the comparative conclusion of no significant difference is unlikely to be substantially altered. Fourth, image quality was assessed using subjective rather than quantitative metrics, as SNR and CNR measurements were difficult to obtain reliably on the small-caliber optic nerve; we therefore used structured visual scoring. Fifth, despite blinding and randomized reading order, the inherent image characteristics of T1-CUBE-DLS may have been recognizable to experienced readers, potentially introducing bias in qualitative assessment. Sixth, enhancement was assessed using both pre and post-contrast images, reflecting routine practice; although pre-contrast images may show intrinsic nerve signal abnormalities, pathological enhancement was determined by consensus interpretation of post-contrast images with side-by-side comparison to baseline. Seventh, etiological classification was based on clinical and serological findings during hospitalization; some patients classified as idiopathic demyelinating ON may later convert to MS or other demyelinating diseases, so this classification reflects baseline categorization rather than eventual diagnostic confirmation. Eighth, this was a single-center study performed on a 3-T GE system using a vendor-specific deep learning framework and a fixed acceleration factor; the results may not be generalizable to other vendors, field strengths, or acceleration factors, and multicenter validation is needed.

## 5 Conclusion

In conclusion, T1-CUBE-DLS reduced acquisition time by 36.2% compared with standard PI-accelerated T1-CUBE, with no significant difference in diagnostic accuracy detected, while providing improved optic nerve clarity, visual conspicuity, and inter-observer consistency in lesion measurement. T1-CUBE-DLS may therefore represent a potentially practical and efficient alternative for assessing acute ON, warranting further evaluation in larger, multicenter settings.

## Data Availability

The original contributions presented in the study are included in the article/supplementary material, further inquiries can be directed to the corresponding authors.
